# Parents’ versus Grandparents’ Attitudes about Childhood Vaccination

**DOI:** 10.3390/children9030345

**Published:** 2022-03-02

**Authors:** Nataša Skitarelić, Marija Vidaić, Neven Skitarelić

**Affiliations:** 1 Department of Health Studies, University of Zadar, 23000 Zadar, Croatia; vidaicmarija@gmail.com (M.V.); nskitarelic@unizd.hr (N.S.); 2 Zadar General Hospital, 23000 Zadar, Croatia; 3 Medicine Faculty, University of Rijeka, 51000 Rijeka, Croatia

**Keywords:** vaccination, vaccines, children, participants, attitudes

## Abstract

Background: We investigated and compared practices and attitudes about childhood vaccination between young parents and their parents and identified influences and sources of information in the County of Zadar, Croatia. Methods: This research was conducted in six general practice and paediatric medical clinics. It included 300 volunteers, including 150 younger parents and 150 older grandparents. Information was collected with a survey questionnaire. The survey data were statistically processed. Results: The 300 participants were divided into 2 groups. Most of the respondents were married, employed, had a high school education, and had a good economic status, often with two children and living in the city. Generally, the attitude towards vaccination was positive. Healthcare workers made the most important influence on the decision for vaccination. The younger age group was significantly affected by social networks and the internet and wanted more information. They were afraid of the adjuvants in vaccines. The older respondents held that vaccination must be legally regulated and did not believe the anti-vaccine media headlines. Conclusions: Our respondents had positive attitudes towards childhood vaccination, noticed the benefits of vaccinating children, and held that untreated children represent a risk for the community. They were well informed and satisfied with the collaboration with medical professionals, although the media and social networks had some impact on attitudes.

## 1. Introduction

Vaccination is an extremely important public health measure that significantly reduced the morbidity and mortality from various infectious diseases in the last sixty years, both in the world and in Croatia. It is one of the important preventative measures to achieve individual and collective immunity. The World Health Organization (WHO) has adopted the “Global Vaccination Strategy” and proclaimed the period from 2011 to 2020 as a “decade of vaccination” [1]. An immunisation plan has been adopted at the European level as the “European Action Plan 2015–2020” to promote vaccination as the most important health care priority that should be included in the regular health system [2].

Although the immunisation project protects more children’s lives than ever before, almost 19.5 million children do not receive even the most basic vaccines, making these children vulnerable to dangerous diseases [3]. More than 1.5 million children die from vaccine-preventable diseases annually in the world [4].

The Childhood Vaccination Program in Croatia is based on mandatory vaccinations, purchased by the state, free of charge. Each year the program is announced by the Minister of Health based on the recommendations by the Croatian National Institute of Public Health. Children in Croatia have been compulsorily vaccinated against eleven different infectious diseases: diphtheria, tetanus, pertussis, poliomyelitis, TBC, measles, rubella, parotitis, hepatitis B, and diseases caused by *Haemophilus influenzae type B* [5]. Since the year 2019, vaccination against pneumococcal diseases also became mandatory [1].

According to the annual report of the Zadar Institute for Public Health for the year 2018, the vaccination coverage of children under 14 years of age in Zadar County was between 92 and 98% for all compulsory vaccines [6], which shows the good state of childhood vaccination. Very similar data are valid for the year 2019 [7]. 

Anti-vaccination movements have been around as long as the usage of vaccinations. The first opponents, anti-vaccine activists, leagues, demonstrations, pamphlets, and movements appeared in the second half of the 19th century. Today, the media, the internet, portals, and social networks are full of anti-vaccine advocates. Recently, in light of the COVID-19 pandemic and the efforts to create a new vaccine, anti-vaccine activists and opponents are emerging, and regular vaccination of children is neglected in many places [8,9,10,11,12,13].

The goal of our research was to investigate the practice and attitudes of young parents of childhood vaccination and compare them with the practice and attitudes of their parents. Moreover, our aim was to identify the sources of information and influence on opinions about childhood vaccination in the County of Zadar, Croatia.

We set these goals precisely because of the strengthening of the anti-vaccine activities in recent decades and the occasional outbreaks of minor disease epidemics among children involving diseases for which children can be vaccinated. All of this is even more visible after the start of the COVID-19 pandemic.

## 2. Materials and Methods

About 170,000 people live in Zadar County, of which 15.78% are children from 0–14 years [14].

Our research was conducted in six general practice and paediatric medical clinics in Zadar County from March to June of 2019. It included 300 volunteers, 150 younger and 150 of older ages. The average age of the younger group of participants was 31.5 years and the average age of the older group was 59.6 years. They completed an anonymous survey questionnaire with 35 questions with answers. The two groups of participants were considered to have voluntarily agreed to participate in the survey if they returned a completed questionnaire that they filled out during visits to paediatric clinics and family medicine clinics. With the survey questions we investigated the sociodemographic characteristics, attitudes, experiences, and knowledge about vaccination from our sample population. The survey data obtained during the test were statistically processed. The statistical data processing included primarily descriptive statistics (frequency analysis and basic descriptive parameters) and interference nonparametric statistics (χ^2^-tests). The licensed software statistical package STATISTICA 13 was used. Values of *p* < 0.05 and *p* < 0.01 were considered statistically significant. The research protocol was approved with number 01-5653/2018, on 28 November 2018, by the Ethics Committee of the Zadar County Health Centre, Zadar, Croatia, EU.

## 3. Results

The study included 300 randomised participants, Caucasians, parents, and grandparents, in 6 general practice and paediatric medical clinics in Zadar County, Croatia, from March to June of 2019.

### 3.1. Sociodemographic Data

Sociodemographic data are shown in Table 1.

Sociodemographic data showed that the majority of the respondents were women (79.33%, *n* = 238), married (90.66%, *n* = 272), employed (71.66%, *n* = 215), with a high school education (92.66%, *n* = 278), and a good economic status (79.66%, *n* = 239), often with at least two children (74.99%, *n* = 225) and living in the city (65%, *n* = 195). 

### 3.2. General Attitudes of Participants about Childhood Vaccination

The results showed that most participants were vaccinated in childhood, about 93.66% (*n* = 281) of them, and they were highly satisfied with their parent’s decision about that. Generally, the attitude towards childhood vaccination was positive. 

The results show that 90.33% of our participants (*n* = 271) decided to vaccinate their children according to the immunization programme, 8% of children (*n* = 24) were partially vaccinated, and 1.66% (*n* = 5) of them were not vaccinated. Of those who did not decide to vaccinate their children, three of them did not vaccinate their children because they were afraid of the risk of vaccination, while two of them considered vaccination unnecessary.

When we asked all our respondents “Do you think that childhood vaccination is a useful procedure?”, a high percentage of them (89%, *n* = 267) gave a positive answer that vaccination is useful for their children, and only 1.66% (*n* = 5) of them thought it is not useful, while 9.33% (*n* = 28) of participants did not know if vaccination is a useful procedure.

The respondents were divided into two groups, a younger group (average age 31.5 years) and an older group (average age 59.6 years). The older group consisted of grandparents that were the parents of the members of the younger group. We were interested to compare the similarities and differences in opinion and attitudes about vaccination between the groups, differences in the amount and sources of information about childhood vaccination, and to detect who most influenced the decision to vaccinate children. We wanted to examine differences in opinion and attitudes across generations. For some questions, we could not compare the groups because the distribution of the given answers did not meet the minimum statistical requirements.

### 3.3. Comparison of Opinions and Attitudes about Vaccination between the Younger and Older Groups of Participants

**Figure 1:**
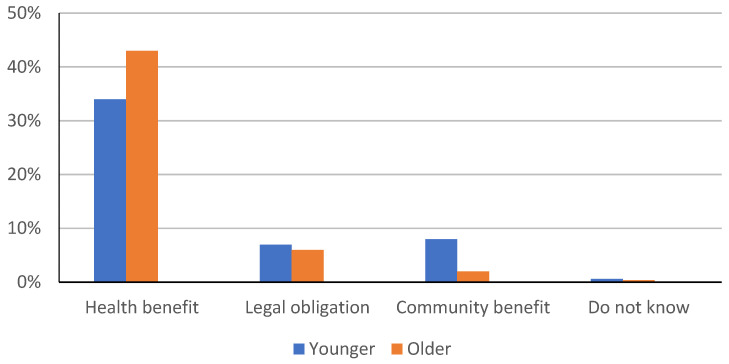
A comparison of the reasons for vaccinating children between the groups of respondents.

The results in Figure 1 show a comparison among the groups of the reasons why respondents choose to vaccinate their children.

Most of the participants from both groups decided to vaccinate their children for the benefit of the children’s health, 75.85% of them (*n* = 223), while 12.93% (*n* = 38) of the respondents opted for vaccination due to the legal obligation. It was observed that the older group preferred children’s health benefits as a reason for vaccination for 9.19% more than the younger group. On contrary, 6.12% more of younger respondents decided to vaccinate their children for the benefit to the community.

In Table 2, we show whether our participants were afraid of vaccination or had doubts about it and the reasons for these feelings.

When asked whether there is a suspicion or fear of childhood vaccination and whether there is a reason for it, 46.33% (*n* = 139) of all participants answered that they do not suspect or fear vaccination. So, after all, a slight majority of our respondents are afraid of vaccination. The analysis shows that there is a statistically significant difference between the groups. At the level of *p* < 0.01 (χ^2^ = 44.16), the younger group is more afraid. We saw that in the older group of participants the most common answer was “I do not doubt or I am not afraid”, but, on the contrary, the younger group gave the same percentage of answers for “I am not afraid” and “I am afraid of vaccine aids”. So, the younger group is more afraid. The most common reasons for fear, in general, were the presence of adjuvants in the vaccine, in 18.0% (*n* = 54) of all participants, but also the vaccine itself, in 9.67% (*n* = 29) of the participants, followed by a needle prick and the stress it causes. From these results, it could be concluded that older respondents have more confidence in the health care system and the justification of vaccination.

### 3.4. Differences between Groups in Impact, Quantity, and Sources of Information about Childhood Vaccination

**Figure 2:**
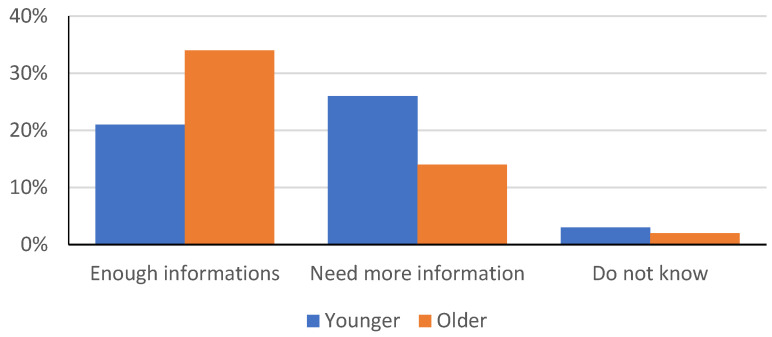
Satisfaction with the quantity of information.

The results of answers to the question “Do you think you have enough information about vaccination and vaccines?” are presented in Figure 2.

When asked if they were satisfied with the amount of information about vaccination and vaccines, as many as 55.0% (*n* = 165) of the participants believed that they were sufficiently informed about vaccinations and vaccines. However, 40.0% (*n* = 120) of the participants would like to receive more information. By comparing the groups, there was a significant difference between the groups, at the level *p* < 0.01 (χ^2^ = 21.61). More participants from the older group believed they were sufficiently informed, while younger ones would like to have more information.

When we compared the answer to the question “Do you think you are sufficiently informed about vaccination and vaccines?” of all respondents, concerning different sociodemographic variables (gender, education level, employment status, household income, and residence) there were no statistically significant differences in responses to the examined sociodemographic characteristics.

Table 3 shows the most important sources of information on vaccination and vaccines in our respondents.

To the question “Who or what is the most important source of information about vaccine and vaccination?”, out of all participants 66.0% (*n* = 198) answered that the most important sources of information about vaccination are healthcare professionals. Social networks and the internet, were shown to have a significant impact on 15.67% (*n* = 47) of respondents. There was a statistically significant difference between the groups, *p* < 0.01 (χ^2^ = 35.9). The analysis shows that social networks and the internet had a significantly greater impact on younger participants, 12.33% (*n* = 37) of them, while healthcare workers had the greatest impact on older participants.

To the question “Who most influenced your decision to vaccinate your child?” our respondents in a high percentage of 82.37% (243 of them) answered that the medical staff had the strongest influence on the decision to vaccinate children. A comparison of the age groups did not show a statistically significant difference.

Moreover, the results showed that our respondents are satisfied, 84.33% or 253 of them, with the support of health professionals in deciding to vaccinate children, while a very small number are dissatisfied, only 2.33% (7 of them).

When asked about the trust of respondents in the selected medical team, as many as 77.0% (*n* = 231) of the respondents fully trust the selected team, while 22.0% (66 of them) believe but want other opinions. Only three respondents do not trust the opinion and advice of the selected doctor about vaccination.

**Figure 3:**
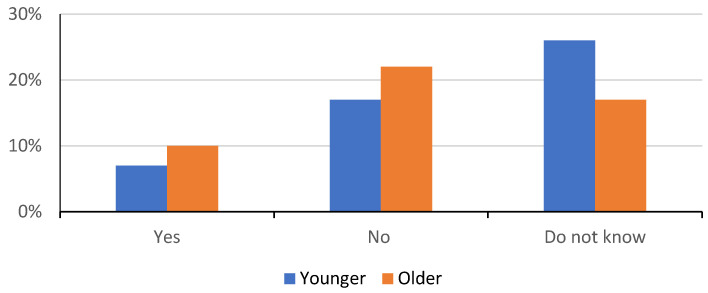
Respondent’s attitudes about anti-vaccination media headlines.

Figure 3 shows the attitudes of our respondents about whether or not they believe in anti-vaccination media headlines.

Moreover, as seen in Figure 3, we obtained results that showed that 17.45% of all respondents, 52 of them, believe in the accuracy of anti-vaccination media headlines, while 43.0% (*n* = 129) of the participants were unsure of their veracity. Only 39.66% (*n* = 119) of the respondents do not believe in anti-vaccination headlines. There was a statistically significant difference between the groups; the older group generally does not believe in anti-vaccination media articles, while the younger participants are significantly more in the category of uncertainty, *p* < 0.05 (χ^2^ = 8.9).

When we compared the answer to the question “Do you believe in the accuracy of anti-vaccination writings?” in all respondents with respect to different sociodemographic variables (age, gender, level of education, and employment status), there were no statistically significant differences in the responses with regard to the examined sociodemographic characteristics. Statistical significance was shown only with respect to household income and place of residence (*p* < 0.01). Those who have middle and higher incomes and reside in the city, believe in the anti-vaccination writings significantly less.

### 3.5. Differences between Groups in Opinions about the Benefits and Legal Obligation of Childhood Vaccination

When asked “Who benefits the most from vaccination?”, the majority of the participants, 224 of them (74.67%) answered that only the child benefits the most from vaccination, and as many as 34 of them (11.33%) answered that the pharmaceutical industry benefits the most. There was a statistically significant difference between the two groups of respondents; the younger group gave more answers in the categories “pharmaceutical industry” and “community, state” while the older group believes that the greatest benefit from vaccination is received by the children themselves, *p* < 0.05 (χ^2^ = 12.64). Therefore, although a large percentage of respondents believe that a child benefits most from vaccination, there is a statistically significant difference in attitudes between groups.

More than half of the participants, 54.33% (*n* = 163) of them, believe that vaccination is the most important reason for the disappearance of certain diseases in the population, while 15.0% (*n* = 45) of them do not think so. Even 30.66% (*n* = 92) of them are not sure about that. Moreover, 45.0% (*n* = 135) of our participants believe that children should not be vaccinated against such diseases, and only 17.33% (*n* = 52) of them think that children should still be vaccinated. The other 37.67% (*n* = 113) of participants have no opinion about that question.

The answers to these two questions proved to be contradictory, suggesting a possibly lower level of information and knowledge about the importance of vaccination coverage.

On the question “Do you think that vaccination should be a legal obligation?” most respondents, 55.67% (*n* = 167) of them, believe that vaccination should be a legal obligation, while others do not consider it or are not sure. The older respondents, to a greater extent, believe that vaccination should be a legal obligation, and the younger respondents, to a much greater extent, believe that it should not or are unsure, *p* < 0.01 (χ^2^ = 9.31). 

## 4. Discussion

Our research showed that in Zadar County, Croatia, respondents have a positive attitude about vaccination, in general. The vast majority expressed a positive attitude towards their vaccination and are pleased that their parents had vaccinated them. Moreover, the children of our respondents are vaccinated in almost the same high percentage. Similar results were found in the Croatian studies [15,16] and some other studies [3,17,18,19,20,21] where researchers noted a very positive parents’ attitude about childhood vaccination and a high vaccination coverage rate. Only 1.66% of our respondents did not vaccinate their children. Such results are indicative of our respondents’ awareness of the benefits of vaccination. Likewise, only a minority of our respondents decided to vaccinate their children due to a legal obligation. It is important to note that almost all vaccinated children were vaccinated according to the Compulsory Vaccination Calendar without the occurrence of complications or difficulties after vaccination. This may be one of the reasons that have contributed to a positive attitude towards vaccination. 

Our study showed, when examining the sociodemographic characteristics, that the majority of respondents had a high school education and a good economic status, which is also associated with positive attitudes about vaccination. Some authors found that parents with higher educational levels are less worried about vaccine safety and have greater confidence in physicians [21], while, on the contrary, some other authors showed that children of university-educated parents had a lower probability of being vaccinated [21]. 

The most significant influence on vaccination decision making in our respondents was health professionals. A study from Sicily also showed that vaccination information obtained from a family paediatrician is significantly associated with the adoption of recommended vaccines by parents, and the most frequent factor influencing parents’ decision about childhood vaccinations was advice from doctors [22]. It is interesting for our respondents that they consider that childhood vaccination is a normal procedure and do not think about it too much, which can be interpreted as the result of a good education, cooperation, and the confidence of parents in health professionals. Moreover, the vast majority of respondents are very satisfied with the collaboration with their primary care team, which they trust almost completely. Respondents who did not vaccinate their children decided after speaking with family and did not follow the recommendations of the professionals. A Spanish study showed that although parents had doubts and thought that vaccines could be harmful, a high percentage of those parents had their children vaccinated. Those results emphasize the importance of health professionals providing adequate information to parents to avoid an increase in negative attitudes to vaccination [21].

When we asked our sample population about suspicion or fear about vaccination and whether there is a reason for it, 46.3% of all participants answered that they do not suspect or fear vaccination. Obviously, a slight majority of our respondents are afraid of vaccination but still accept it. Similar results were shown by Raof [23]. Although most respondents accepted vaccination, younger respondents reported significantly more frequent fear of adjuvants in vaccines, but also the vaccine itself, a needle prick, and the stress it causes, indicating the influence of social networks and media. This is also a consequence of the strengthening of anti-vaccine activities in recent decades [9]. Specifically, anti-vaccine activists often emphasize vaccine supplements, excipients, preservatives, mercury, and thimerosal as probable causes of adverse events and illness after vaccination. For example, one study highlights the highly causal link between autism and aluminium in vaccines [24]. Such theories have been rejected because many recent studies have not found sufficient evidence to conclude there is a causal link between aluminium and autism. From our results, it could be concluded that older respondents have more confidence in the health care system and the justification of vaccination, while younger respondents are more influenced by the media and the internet.

Examining the main sources of vaccination information, we found that most of our respondents cited health professionals as the most important sources. However, there was a statistically significant difference between the age groups. Namely, younger respondents would like additional information, and most of all, they go to social networks and the internet. The examined sociodemographic characteristics did not show statistically significant differences among the age groups in that question. A survey from Serbia and the Netherlands showed very similar results to ours [3,25], while another study from Turkey showed that a significant number of their participants stated that the information about childhood vaccinations is unreliable or they doubted the credibility of the information [26].

Other studies from Europe and USA showed that social networks and the internet are very often used as a source of vaccination information, and advice from friends, family, and colleagues or specific life events, family history, religion, and less scientific facts play roles in the formation of attitudes toward vaccination [27,28]. 

As the media and the internet have a great influence on the formation of public opinion today, in our research, we investigated the opinion of the respondents about their belief in media articles. Only a minority of our respondents believed in the accuracy of anti-vaccination media reports, while less than half were uncertain about the credibility of the inscriptions, especially those of the younger age group. According to recent data, more than 50% of older people use the internet and social networks on daily basis [29]. Despite that, the older group of our participants generally does not believe in anti-vaccination media articles, while younger participants are significantly more in the category of uncertainty and showed a statistically significant difference. In addition, those residing in the city and with a good socioeconomic status do not trust the vaccination insignia much more. The older population is expected to have an opinion based on life experience, while younger respondents have yet to formulate their views.

The majority of the respondents in our study expressed their satisfaction with the support of health professionals. An Australian study highlighted the interaction of parents with healthcare professionals as a key factor in achieving good vaccine coverage among their subjects [30].

Although the majority of our respondents consider that the child benefits from vaccination alone, a smaller proportion of respondents cited the pharmaceutical industry and the community, mostly members of the younger age group. There was a statistically significant difference between the two groups of respondents; the younger group gave more answers in the categories “pharmaceutical industry” and “community, state”, while the older group believes that the greatest benefit from vaccination is received by the children themselves. Therefore, although a large percentage of respondents believe that only a child benefits most from vaccination, there is a statistically significant difference in attitudes between groups.

Such answers suggest the influence of the media, social networks, and the anti-vaccine movements. A Dutch study showed similar results, highlighting that parents believe that the pharmaceutical industry is influenced by government decisions on vaccinations and vaccines [28]. 

Most of our respondents believe that vaccination should be a legal obligation, while a third of respondents believe that vaccination should be a matter of personal choice. However, most respondents would vaccinate their children even if vaccination was not a legal obligation. Similar results have been shown by other studies [3,15,22]. So, more parents agree with the benefits than with barriers to vaccination. 

More than half of our respondents believe that vaccination is the most important reason for the disappearance of certain diseases in the population and that non-vaccinated children present a risk to the wider community. However, at the same time, less than half of the respondents found that children do not need to be vaccinated against diseases that have disappeared in the population. This answer interestingly contradicts the previous answer. This can be interpreted as the result of the low level of awareness about that question. Namely, for a disease to be eradicated in the population, it is important to maintain a high vaccine coverage against the disease.

Our study has some notable limitations. First of all, there is a limited sample size included in the study. Furthermore, the participants share very similar sociodemo-graphic characteristics, which could affect the representativeness of the study.

Despite the limitations in our study, it clearly shows that strong recommendations from healthcare professionals are the main and most influential source of vaccination information for most people. They need to support families, educate them, and try to vaccinate as many children as possible. So, no doubt, good, clear, and permanent communication and collaboration between healthcare professionals and parents are necessary. It is the most important thing and the cornerstone for the confidence between healthcare professionals and public opinion. That will prevent the negative influence of some media headlines and anti-vaccine activists, which have been loud for the last few decades, especially now in the COVID-19 pandemic.

## 5. Conclusions

From all of the above, we can conclude that the Zadar County respondents, generally, have a positive attitude towards childhood vaccination and notice the health benefits. Although they have some fear and suspicion about vaccination and the adjuvants in vaccines, especially evident in the younger population, they hold that untreated children represent a risk for the community. They are satisfied with the collaboration, trust the healthcare professionals, and are sufficiently informed, although they want even more detailed information. The younger population would like to be even better informed, but they are more influenced by the social networks and the internet, which is understandable given the modern communication capabilities. There are differences in attitudes and practices between the generations, which this study was designed to identify.

We can confirm that advice from healthcare professionals is the main and most influential source of vaccination information for most people. It is up to healthcare professionals to keep abreast of current vaccination trends, to be well informed, and to pass on that information to their users for the mutual satisfaction and well-being of the wider community.

## Figures and Tables

**Table 1:** Sociodemographic data of the participants.

Characteristics	N (%)
Gender	
Women	238 (79.33%)
Men	62 (20.67%)
Employment	
Full-time	167 (55.66%)
Part-time	48 (16.00%)
Unemployed	54 (18.00%)
Retired	31 (10.33%)
Marital status	
Married	272 (90.66%)
Unmarried	9 (3.00%)
Divorced/widow	19 (6.33%)
Education	
Elementary school	22 (7.33%)
High school	178 (59.33%)
College/University	100 (33.33%)
Number of children	
1	75 (25.00%)
2	150 (50.00%)
3	56 (18.66%)
≥4	19 (6.33%)
Monthly income	
≤EUR 600	61 (20.33%)
EUR 601–EUR 1200	176 (58.66%)
>EUR 1200	63 (21.00%)
Residence	
Village	105 (35.00%)
Town	195 (65.00%)

**Table 2:** Reasons for fear of vaccination.

	Vaccine	Adjuvants	Puncture Stress	All of the Above	Other	Not Afraid
Younger	15 (5%)	40 (13.33%)	16 (5.33%)	27 (9%)	9 (3%)	43 (14.33%)
Older	14 (4.67%)	14 (4.67%)	9 (3.00%)	10 (3.33%)	7 (2.33%)	96 (32.00%)
Total	29 (9.67%)	54 (18.00%)	25 (8.33%)	37 (12.33%)	16 (5.33%)	139 (46.33%)

**Table 3:** The most important source of information about vaccination and vaccines.

	Family	Friend	Social Network	Medical Staff	Other
Younger	14 (4.67%)	7 (2.33%)	37 (12.33%)	88 (29.33%)	4 (1.33%)
Older	29 (9.67%)	0 (0%)	10 (3.33%)	110 (36.67%)	1 (0.33%)
Total	43 (14.33%)	7 (2.33%)	47 (15.67%)	198 (66%)	5 (1.67%)

## Data Availability

The data presented in this study are available on request from the corresponding author.
